# Value of information analysis for pandemic response: intensive care unit preparedness at the onset of COVID-19

**DOI:** 10.1186/s12913-023-09479-4

**Published:** 2023-05-13

**Authors:** Peter U. Eze, Nicholas Geard, Christopher M. Baker, Patricia T. Campbell, Iadine Chades

**Affiliations:** 1 School of Computing and Information Systems, University of Melbourne, Victoria, Australia; 2 School of Mathematics and Statistics, University of Melbourne, Victoria, Australia; 3 Melbourne Centre for Data Science, University of Melbourne, Victoria, Australia; 4 Centre of Excellence for Biosecurity Risk Analysis, University of Melbourne, Victoria, Australia; 5 Department of Infectious Diseases, University of Melbourne, at the Peter Doherty Institute for Infection and Immunity, University of Melbourne, Victoria, Australia; 6 Melbourne School of Population and Global Health, University of Melbourne, Victoria, Australia; 7 CSIRO Land and Water Dutton Park, CSIRO, Brisbane, Australia

**Keywords:** Outbreak preparedness, Value of information, Mathematical modelling, COVID-19

## Abstract

**Background:**

During the early stages of the COVID-19 pandemic, there was considerable uncertainty surrounding epidemiological and clinical aspects of SARS-CoV-2. Governments around the world, starting from varying levels of pandemic preparedness, needed to make decisions about how to respond to SARS-CoV-2 with only limited information about transmission rates, disease severity and the likely effectiveness of public health interventions. In the face of such uncertainties, formal approaches to quantifying the value of information can help decision makers to prioritise research efforts.

**Methods:**

In this study we use Value of Information (VoI) analysis to quantify the likely benefit associated with reducing three key uncertainties present in the early stages of the COVID-19 pandemic: the basic reproduction number ($R_0$), case severity (*CS*), and the relative infectiousness of children compared to adults (*CI*). The specific decision problem we consider is the optimal level of investment in intensive care unit (ICU) beds. Our analysis incorporates mathematical models of disease transmission and clinical pathways in order to estimate ICU demand and disease outcomes across a range of scenarios.

**Results:**

We found that VoI analysis enabled us to estimate the relative benefit of resolving different uncertainties about epidemiological and clinical aspects of SARS-CoV-2. Given the initial beliefs of an expert, obtaining more information about case severity had the highest parameter value of information, followed by the basic reproduction number $R_0$. Resolving uncertainty about the relative infectiousness of children did not affect the decision about the number of ICU beds to be purchased for any COVID-19 outbreak scenarios defined by these three parameters.

**Conclusion:**

For the scenarios where the value of information was high enough to justify monitoring, if *CS* and $R_0$ are known, management actions will not change when we learn about child infectiousness. VoI is an important tool for understanding the importance of each disease factor during outbreak preparedness and can help to prioritise the allocation of resources for relevant information.

**Supplementary Information:**

The online version contains supplementary material available at 10.1186/s12913-023-09479-4.

## Background

When confronted with an emerging infectious disease, public health managers face the challenge of allocating finite health resources to maximise outbreak preparedness [1, 2]. However, many important characteristics of emerging infectious diseases are unknown in the early stages of an outbreak, which complicates the allocation of funds when it is most urgently needed. Faced with this challenge, public health managers can decide to allocate available resources under uncertainty to manage an outbreak, or invest some of these resources to reduce uncertainty by collecting additional information about the disease outbreak. Under time and resource constraints, strategically collecting the most valuable information is even more critical [3] as obtaining new information can be time consuming and come with both direct and opportunity costs [4, 5]. In the case of an emerging infectious disease, time and resources invested in collecting information could instead be invested directly into outbreak preparedness or interventions. An example of such a decision, relevant in the early stages of the COVID-19 pandemic, was the acquisition of Intensive Care Unit (ICU) beds. While it was clear that increased capacity of health care systems would be needed, yet determining the optimal level of investment was not straightforward. Value of Information (VoI) analysis is a decision tool that helps managers make transparent and accountable decisions when faced with this dilemma of either acting under uncertainty or gathering more information about a given situation before acting [6].

VoI analysis was first developed by Raiffa & Schlaifer [6] in 1961 for application in investment risk analysis. VoI has been applied to ecological and environmental conservation problem-solving and analysis [3, 4, 7–10]. In the field of epidemiology and public health economics, VoI has been applied for the prioritization of information gathering during vaccination and other non-pharmaceutical interventions, and for the assessment of health technology adoption [11–13]. Bradbury et al [12] provides a compelling example of how VoI analysis can inform decision making. In that study, VoI was applied to the analysis of an emergency vaccination campaign for Foot-and-Mouth Disease (FMD) in livestock. There was substantial uncertainty around vaccine efficacy, the time lag between vaccination and conferral of immunity on the animals against the virus, and the daily capacity to deliver vaccines. With some defined uncertainties (expanded into 27 scenarios), management actions and objectives, Bradbury et al [12] found that resolving these uncertainties would lead to an average savings of 55 million pounds among other benefits in reducing the number of infected animals as well as the duration of the epidemic. The implication of this finding is that they could identify the most valuable uncertainty to resolve.

Faced with the COVID-19 pandemic, many questions were raised about the capacity of healthcare systems around the world to cope with emerging disease outbreaks [14, 15]. During the early outbreak of COVID-19 in Wuhan, 75% of deaths resulted from lack of access to mechanical ventilation [16]. Similar experiences in Italy and Singapore showed the importance of ICU preparedness for respiratory disease outbreaks [17, 18]. Increasing the number of ICU beds and other critical care preparations will increase the survival rate of severe COVID-19 cases. Noting that acquiring more ICU beds alone is not sufficient and appropriate management practices are essential to optimum utilisation of hospital resources [17, 19, 20].

Here, we explore how managers can use VoI to decide whether to invest in gathering further information about COVID-19 disease dynamics before purchasing ICU beds or to purchase ICU beds directly without collecting further information. We study how three uncertain parameters relate to ICU demand: the basic reproduction number ($R_0$), case severity (*CS*) and the relative infectiousness of children compared to adults (*CI*). We aim to discover which parameter is the most valuable to enable good decisions around ICU preparedness. We use a dynamic transmission model and a clinical pathways model [21] to quantify potential ICU demand, and then apply the VoI analysis.

## Method

VoI analysis estimates the value to be gained from resolving uncertainty [3, 6]. In this section, we present an application of VoI to the problem of deciding the appropriate level of investment in ICU bed capacity prior to a novel infectious disease outbreak, given uncertainty about local transmission and severity characteristics of the disease. We first define the problem context used as our case study. We present the management objectives in terms of preparing for a COVID-19 outbreak in a resource-constrained setting. We define the management actions that can be chosen to meet the objective(s). We then introduce two VoI methods that assess how resolving transmission and severity uncertainties affects the choice of management actions. We also describe the models used to generate the input data for our analysis.

### Problem context and case study

The World Health Organization Regional Office for the West Pacific (WPRO) monitors the public health performance of about 1.9 billion people across 37 countries in the West Pacific region. Across the WPRO countries, the healthcare system is generally under stress and requires strengthening especially for novel outbreaks [22]. ICU capacity varies significantly across countries in the WPRO region, with a capacity range in some as low as just a few (if any) beds to enable care of severe cases of COVID-19 [14]. Others do have ICU beds but their numbers may be insufficient during a large outbreak. Information such as the characteristics of the population and nature of the outbreak are important features that influence the required number of ICU beds in these countries. Unfortunately, this information is often missing. Here, we use VoI to provide insight into which information is most valuable to prepare for a COVID-19 outbreak in the WPRO region. We considered a typical urban settlement in the Asia-Pacific Islands with an approximate population of 450,000 with 9 ICU beds for COVID-19 (Table 1). This choice corresponds to a relatively high populated settlement within the Asia-Pacific islands. The model inputs for this population are presented in the accompanying Supplementary information (Section 1, Table S1).

**Table 1:** Population-level parameters: A critical care hospital with 18 ICU beds, of which half are assumed to be reserved for COVID-19 patients

S/N	Parameter	Value
1.	Population	450,000
2.	Total ICU Bed	18
3.	ICU Bed for COVID-19	9

### Objective and actions

The first step in formulating VoI analysis is defining the management objective and the actions that will lead to achieving these objectives. This approach is necessary because a piece of new information is only valuable if it will lead to a change in outcomes.

The broad objective is to minimise the health impact of COVID-19 through the optimal provisioning of ICU beds for both COVID-19 and other existing diseases within a population. We translate this broad objective into: *minimising the deficit or excesses in newly acquired ICU beds for COVID-19 outbreak preparation*. The outcome measure is the sum of ICU bed shortfall over the duration of the outbreak. Because increasing the number of ICU beds for COVID-19 also comes at a real cost and an opportunity cost, our objective includes minimising surplus allocation of ICU beds to COVID-19 that could result in an adverse effect on other existing diseases in the given population. Hence, we intend to minimise the absolute difference between any newly acquired ICU beds before an outbreak plus existing ICU beds, and the actual number of ICU beds utilised to contain an outbreak.

We define the actions available to a health manager as various increase options in ICU beds capacity from 0 to 200. We consider only the first five discrete actions available to a decision-maker (using increments of 50 beds):

- $$a_0$$: Do nothing.
- $a_1$: Increase capacity by 50 ICU beds.
- $a_2$: Increase capacity by 100 ICU beds
- $a_3$: Increase capacity by 150 ICU beds
- $a_4$: Increase capacity by 200 ICU beds

The choice of maximum expansion of up to 200 ICU beds is a reflection of the plausible range for a Low-and-medium Income Country (LMIC), based on discussion with health system researchers within the region under study. Further, the idea of ICU bed is not limited to the equipment itself but the personnel and logistics required to operate the ICU beds. Therefore, we limit the actions to the maximum plausible irrespective of actual demand.

There are a range of methods that assist in VoI analysis [3, 6]. The expected value of perfect information (EVPI) quantifies how beneficial it is to resolve all uncertainty before making a decision. The expected value of partial information (EVPXI) estimates the improvement in decision outcomes if information is resolved about one (or more) parameter. Both approaches calculate expected values and therefore assume a risk neutral situation [23].

### Expected value of perfect information

We define a COVID-19 outbreak scenario, *s*, as a combination of values of three epidemiological parameters: case severity *CS*, basic reproduction number $R_0$, and child infectiousness *CI*. These parameters are uncertain and correlate with early factors used in determining the number of patients that may need access to the ICU of a hospital during a COVID-19 outbreak [24].

Given an uncertain scenario $s \in S$, and a set of alternative actions $a \in A$, the EVPI is the difference between the expected outcomes under certainty ($EV_{certainty}$) and uncertainty ($EV_{uncertainty}$) [3, 6].

1 $$\begin{aligned} EVPI = |EV_{certainty} - EV_{uncertainty }| \end{aligned}$$

Because our outcomes are measured as a shortfall (which is a form of health cost instead of health benefit), we use absolute values for the calculation of EVPI to avoid negative values during computation. Under uncertainty, it is assumed that decision-makers will implement the action that minimises the expected cost outcomes under all possible scenarios. Given a number of scenarios, *N*, in a set, *S*:

2a $$\begin{aligned} EV_{uncertainty}= \underset{a}{\textrm{min}} \left\{ \sum \limits _{s=1}^NV(a,s).p_s \right\} \end{aligned}$$

with *V*(*a*, *s*) the cost function that represents the outcome of implementing action *a* in scenario *s*, and $p_s$ is the probability of scenario *s* occurring.

Under certainty, it is assumed that decision-makers will first invest in resolving uncertainty prior to making a decision *a* that minimises the cost of an outbreak scenario.

2b $$\begin{aligned} EV_{certainty}= \sum \limits _{s=1}^N p_s. \underset{a}{\textrm{min}} V(a,s) \end{aligned}$$

We define our cost function *V*(*a*, *s*) such that it incorporates the effect of other diseases requiring ICU beds. For each scenario *s*, and corresponding action *a*, the cost function *V*(*a*, *s*) measures the level of shortfall or excess in ICU beds in preparedness for a COVID-19 outbreak:

3 $$\begin{aligned} V(a,s) = \left\{ \begin{array}{ll} \gamma \left| U(a,s) \right| ,&{} \text {if } U(a,s) < 0\, ; 0 \le \gamma \le 1 \\ U(a,s), &{} \text {otherwise} \end{array}\right. \end{aligned}$$

Where *U*(*a*, *s*) is the utility of an action *a* in scenario *s*, and $\gamma$ is a discount factor that takes value between 0 and 1 to represent the impact on other diseases when newly-acquired ICU beds (*B*(*a*)) are allocated to COVID-19 to the detriment of other non-COVID severe illnesses. In countries where bed allocation to COVID-19 does not cause problems for other existing conditions in the society $\gamma$ should be set to 0. Countries that cannot afford to have any extra allocation to COVID-19 would assign $\gamma = 1$.

We define the utility of an action *a*, in a given scenario *s*, as

4 $$\begin{aligned} U(a,s)= X(s) - B(a) \end{aligned}$$

Where *X*(*s*) is the shortfall in ICU beds required to address an outbreak under scenario *s* and *B*(*a*) is the number of ICU beds acquired while implementing an action, *a*. The utility *U*(*a*, *s*) is optimised when the acquired ICU beds *B*(*a*) exactly matches a possible shortfall in ICU beds *X*(*s*) during an outbreak scenario *s*.

In accordance with our objective, our cost function *V*(*a*, *s*) penalises a shortage of ICU beds for COVID-19 (*U*(*a*, *s*)) but also recognises that excess allocation of ICU beds to COVID-19 to the detriment of other diseases that also require ICU beds ($\gamma |U(a,s)|$) is not an optimal decision.

### Expected Value of Partial Perfect Information (EVPXI)

EVPI determines the value of resolving all uncertainty in a decision process. However, EVPI does not recommend which uncertain scenario to resolve first [3]. This can be useful in cases where EVPI is *high enough* and health managers need to determine which of the uncertain scenarios is most important to resolve. The calculation of EVPXI helps guide such a decision. EVPXI calculates the improvement in expected outcomes when resolving uncertainty about a scenario and is expressed as:

5 $$\begin{aligned} EVPXI = |EV_{certainty(y)} - EV_{uncertainty} | \end{aligned}$$

Where $EV_{certainty(y)}$ corresponds to the expected value when uncertainty about scenario *y* being true or false is resolved. Because we are using a cost function rather than a payoff, we use an absolute value to ensure that a positive value is returned as EVPXI. When computing the expected value of the hypothesis *y*, we have one of two outcomes: either *y* is true (with probability $p_{y = true}$) or it is not (with probability $p_{y = false}$). Formally:

6 $$\begin{aligned} EV_{certainty(y)} = p_{y=true} \min _a V(a,y)+ (1- p_{y=true}) {\sum \limits _{s=1|s \ne y}^N \underset{a}{\textrm{min}} V(a,s).p_{s|y=false} } \end{aligned}$$

where the first term calculates the expected value in the case where scenario *y* is true with probability $p_{y = true}$, and the second term calculates the expected values in the case where *y* is false [3]. If *y* is false, then calculating the expected outcomes requires determining $p_{s|y}=false$, i.e. the re-normalised probability distribution computed for the remaining uncertain scenarios. For example, let $p_s = \{0.2, 0.5,0.3\}$ be our probabilities in three scenarios: $s_1$, $s_2$ and $s_3$. When we resolve $s_1$ to be false, then our probabilities in $s_2$ or $s_3$ change to $p_{s|y = false}=\{0.625,0.375\}$.

### The value of resolving individual parameters

The scenario modelling is useful to estimate the expected outcomes of a management decision. However, from a research perspective, it might be easier to quantify the effect of resolving uncertainty about only one parameter at a time, instead of combined values represented by a scenario. For example, health managers could study only how case severity *CS* being *Low*, *Medium* or *High* affects ICU requirements. To find out which parameter uncertainty is worth reducing, we can reapply the EVPI Eq. (1) . However, we first need to redefine *X*(*s*) in the case where *s* is now explicitly differentiating the contribution of each parameter, i.e. $s = \{s_{R_0},s_{CS},s_{CI}\}$. The value of resolving an individual parameter becomes the marginal expected outcomes over the set of unknown parameters and can be expressed as:

$$\begin{aligned} X(s_{R_0}=x)= {E}_{y \sim s_{CS},z \sim s_{CI}} [X(x,y,z)], with\ x \in \{Low, Medium, High\} \end{aligned}$$

$$\begin{aligned} X(s_{CS}=y)= {E}_{x \sim s_{R_0},z \sim s_{CI}} [X(x,y,z)], with\ y \in \{Low, Medium, High\} \end{aligned}$$

$$\begin{aligned} X(s_{CI}=z)= {E}_{x \sim s_{R_0}, y \sim s_{CS}} [X(x,y,z)], with\ z \in \{Low, Medium, High\} \end{aligned}$$

For example, if we are only interested in studying sub-scenarios in which $s_{CS} \in \{Low, Medium, High\}$, we can compute the expected outcomes for $X_{CS=Low}$, $X_{CS=Medium}$ and $X_{CS=High}$ assuming a uniform distribution over the values of the parameters $R_0$ and *CI*. Specifically, to compute the cost for the sub-scenario where *CS* is *High*, we take an average of the model outputs of nine scenarios where $X_{CS} = High$:

$$\begin{aligned} X(s_{CS}=High)= \frac{ \sum _{x\in s_{R0}, z \in s_{CI}}X(x,s_{CS}=High,z)}{9} \end{aligned}$$

In other words, the term *X*(*s*) in Eq. (4) is replaced with the average value $X(S_{CS}=High)$. Repeat the calculations for $X(S_{CS}= Medium)$ and $X(S_{CS}=Low)$. Hence, for resolving the case severity parameter uncertainty, we are concerned with only three broad scenarios where $CS = (Low|Medium|High)$ . With these scenarios, we compute an EVPI for CS. We then repeat this process for $R_0$ and *CI*. The parameter with highest EVPI will need to be resolved first.

### Infectious disease modelling and simulation

**Fig. 1:**
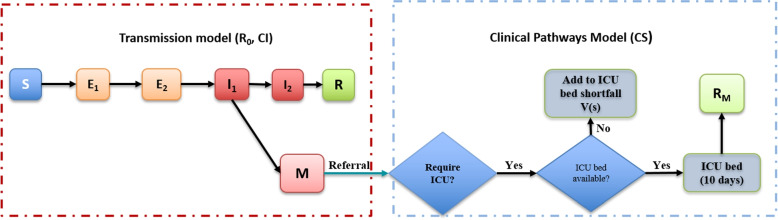
The transmission model and the clinical pathways model: Only infected and managed individuals who do not recover without treatment and who present at the hospital (M) can require an ICU bed. In this study, we consider these managed cases that are likely to require an ICU bed but may not access it. Parameter details are provided in theSupplementary information

We briefly describe the model employed to compute the shortfall of ICU beds *X*(*s*) for each of the 27 outbreak scenarios defined by three parameters, each stratified into three value space. We use a combined transmission model (TM) and clinical pathway model (CPM) to estimate the cost of a COVID-19 outbreak in terms of the shortfall in ICU beds and explored how this cost changes for different combinations of disease parameters $R_0$, *CS*, and *CI*. We build on previously published Susceptible-Exposed-Infectious-Recovered (SEIR) and clinical pathway models [21] developed to inform intervention decisions for both Influenza and COVID-19 outbreaks [25] to compute *X*(*s*) (Fig. 1).

The SEIR model compartmentalises a population into susceptible, exposed, infected or recovered segments. It uses differential equations to represent how the number of people in each compartment changes over time based on some model parameters including $R_0$ [26] and relative infectiousness of children (*CI*). The clinical pathway model, on the other hand, applies to individuals who become infected and also present themselves for medical attention. Patient presentation at different levels of severity is represented by the *CS* level parameter. The model’s population is age-structured such that for each scenario, the proportion of individuals in an age group with severe infection differs by age group. For example, the age ranges $0-19$ in ($CS_{Low}$) will have 0.062% of its population having a severe infection. The proportion for the age range 60 - 69 years for $CS_{Low}$ is 15.4% but for $CS_{Medium}$, the proportion increases to 66% for this age range 60-69 years. Details of these proportions are provided in Section 1 of the Supplementary information.

We selected parameter combinations as follows. We first stratified each parameter range into three categories: *Low*, *Medium* or *High* (Table 2) based on observations made during the early stages of the COVID-19 pandemic (see Section 1 in Supplementary information). To ensure efficient coverage of parameter space, we selected specific parameter values from each of the nine possible combinations of these categories using Latin Hypercube Sampling (LHS)[27]. Hence, when simulating a parameter value from the *Low*, *Medium* or *High* category, we first divided the category into 200 subintervals of equal probability and then selected a value uniformly at random from each of those subintervals, yielding 200 parameter values within a category. This approach helped us to integrate uncertainty within the decision process.

In Table 2, the value of *CS* determines the proportion of cases that become severe within the population. Only severe cases get hospitalized and only a fraction of the hospitalized cases end up in the ICU unit. Hence, the higher the value of *CS*, the higher the proportion of people that are likely to require an ICU bed.

**Table 2:** Qualitative and Quantitative Stratification of Input Parameters studied. The LHS Monte-Carlo sampling framework was used for sampling in each interval

Parameter	Low value	Medium value	High value
Basic Reproduction Number ($R_0$)	$1 - <1.5$	$1.5 - <2.5$	2.5 - 3.0
Case Severity (CS)	1	2	3
Child infectiousness (CI)	$0 - < 40\%$	$40 - < 70\%$	70 - 100%

A summary of the models applied to this study is shown in Fig. 1.

We define $R_0$ as:

7 $$\begin{aligned} R_0 = \lambda * \kappa * d \end{aligned}$$

where $\lambda$ is the probability of getting infected per contact between an infectious and a susceptible individual, $\kappa$ is the contact rate between infectious and susceptible individuals, and *d* is the duration of infectiousness of infected individuals before recovery or death.

The shortfall in ICU beds (after the available ICU beds are exhausted) required per outbreak scenario, *X*(*s*), was estimated through simulations. During the TM and CPM simulations, the values of $R_0$, *CS* and *CI* were sampled 1000 times within their uniform range of values. However, during the VoI simulations, the expert beliefs in Table 3 were used to scale the output of the models (*X*(*s*)) for each of the 27 scenarios presented in Table 4.

### Sensitivity analysis on prior information

In the previous sections, we have assumed that each scenario was assigned a prior probability of occurrence $p_s$. This prior information influences the result of value of information analysis. In the absence of prior knowledge, we assumed equal chance over the values of the unknown parameters ($R_0, CS, CI$) being *Low*, *Medium* or *High*.

To study the influence of these probabilities $p_s$ on the value of information, we also defined unequal chances over the parameter categories. We considered priors where we had a “Strong belief” that all the parameters are either *Low*, *Medium* or *High*. Table 3 provides the probability distribution over each of the parameter categories for these three priors. For example, for the “belief in low outbreak”, we will assume that the scenario corresponding to $s_{R_0}=s_{CS}=s_{CI}=Low$ will have the highest probability $p(s_{R_0}=Low) = p(s_{CS}=Low) = p(s_{CI}=Low) = 0.5$.

**Table 3:** Beliefs in the nature of Outbreaks: The prior beliefs of 0.5 for all $p(s_{R_0})$, p($s_{CS}$) and $p(s_{CI})$ in each category (*Low*, *Medium* or *High*) defines a strong belief that an outbreak will be low, mild or severe. The baseline belief (not shown in this table) is *No prior knowledge* where an *expert* has no prior knowledge about the possible value range for $R_0$, *CS* and *CI*. In the baseline belief, the probability that the parameters assume *Low*, *Medium* or *High* value ranges are all equal: $p(s_{R_0}) = p(s_{CS}) = p(s_{CI}) = 0.33^+$

	Belief in low outbreak			Belief in medium outbreak			Belief in severe outbreak		
*category*	$p(s_{R_0})$	$p(s_{CS})$	$p(s_{CI})$	$p(s_{R_0})$	$p(s_{CS})$	$p(s_{CI})$	$p(s_{R_0})$	$p(s_{CS})$	$p(s_{CI})$
*Low*	0.5	0.5	0.5	0.3	0.3	0.3	0.2	0.2	0.2
*Medium*	0.2	0.2	0.2	0.5	0.5	0.5	0.3	0.3	0.3
*High*	0.3	0.3	0.3	0.2	0.2	0.2	0.5	0.5	0.5
**1.0**	**1.0**	**1.0**	**1.0**	**1.0**	**1.0**	**1.0**	**1.0**	**1.0**	**1.0**

Assuming independence, the joint probability, $p(R_0,CS, CI)$ that an epidemic belongs to one of the 27 outbreak scenarios is the combinations of Low, Medium and High parameter values:

8 $$\begin{aligned} p(s_{R_0},CS, CI)=p(s_{R_0}) \times p(s_{CS}) \times p(s_{CI}) \end{aligned}$$

As an example, a belief that a future outbreak will have Low $R_0$, High Severity and Low Child Infectiousness has the probability:

$$\begin{aligned} p (s_{R_0}=Low, s_{CS}=High, s_{CI}=Low) = 0.5 \times 0.2 \times 0.3 = 0.03 \end{aligned}$$

Hence, our sensitivity analysis represents the 27 hypothetical scenarios of a novel disease outbreak based on all possible combinations of the 3 uncertain parameters under consideration, each grouped into high, medium or low values.

## Results

### Cost of an outbreak from simulations

The first result we provide is the shortfall in ICU beds, *X*(*s*), for each scenario $s_1$ to $s_{27}$ obtained through simulations of the transmission and clinical pathway models (Table 4). The *X*(*s*) values reported in this table are the $95^{th}$ percentile of the maximum ICU bed shortfall for all the simulations per scenario. The choice of $95^{th}$ percentile guarantees that 95% of ICU requirement per outbreak scenario is met.

**Table 4:** Shortfall in ICU beds, *X*(*s*) for each scenario: The shortfall in ICU beds is the direct output from the transmission model after each of the scenarios is run. Each scenario is a unique combination of the outbreak parameters: transmissiblity ($R_0$), case severity (*CS*) and child infectiousness (*CI*)

	Parameter States			Shortfall in ICU bed
Scenario ID	$R_0$	Case Severity (*CS*)	ChildInf (*CI*)	*X*(*s*)
1	Low	Low	Low	0
2	Low	Low	Medium	0
3	Low	Low	High	0
4	Low	Medium	Low	0
5	Low	Medium	Medium	0
6	Low	Medium	High	0
7	Low	High	Low	0
8	Low	High	Medium	0
9	Low	High	High	345
10	Medium	Low	Low	0
11	Medium	Low	Medium	0
12	Medium	Low	High	0
13	Medium	Medium	Low	0
14	Medium	Medium	Medium	29
15	Medium	Medium	High	74
16	Medium	High	Low	552
17	Medium	High	Medium	1075
18	Medium	High	High	1406
19	High	Low	Low	0
20	High	Low	Medium	0
21	High	Low	High	0
22	High	Medium	Low	46
23	High	Medium	Medium	102
24	High	Medium	High	157
25	High	High	Low	1389
26	High	High	Medium	1584
27	High	High	High	1485

The scenarios where $X(s) = 0$ imply that existing ICU beds are enough to take care of any severe outbreak. It could also mean that the outbreak is not severe enough to cause critical illness. So, there is no shortfall in ICU beds that requires additional purchase or assignment to COVID-19. When case severity is *Low*, $X(s)=0$ irrespective of the value of $R_0$ and child infectiousness. *X*(*s*) is also zero when case severity is *Medium* but $R_0$ is *Low*; child infectiousness will not matter. In the other extreme, when case severity is *High* and $R_0$ is also *High*, $X > 1000$ ICU beds irrespective of the value of child infectiousness. A decision tree is provided in Fig. S3 of supplementary information to show the combinations of parameters and how large the *X*(*s*) can be in terms of excess ICU bed demand.

### Expected value of perfect information

The expected value of perfect information (EVPI) depends on the prior information on the most likely scenario (beliefs) and the impact of other diseases (denoted by $\gamma$). Given that $\gamma$ will be country-dependent, we vary the value of $\gamma$ to interpret our results.

**Fig. 2:**
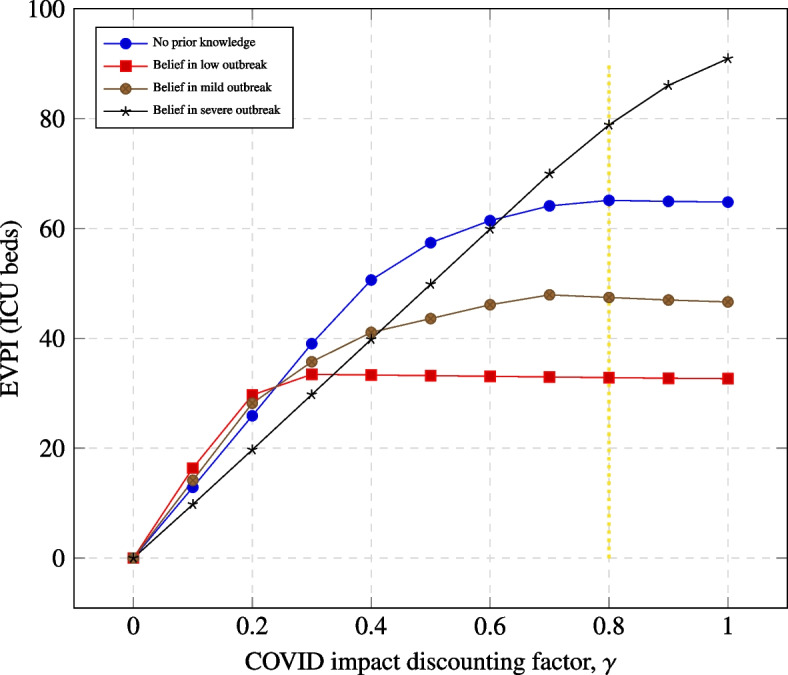
EVPI varies with discount factor $\gamma$ and prior belief in the strength of an outbreak. EVPI reaches a peak at $\gamma = 0.8$ (yellow dashed line) except for the scenarios with *belief in severe outbreak*. The factor, $\gamma$ is a region-specific factor that accounts for the impact of COVID-19 on other existing diseases in that region

Under *No prior knowledge* (Fig. 2 - blue curves), EVPI varied from 0 to 65 ICU beds depending on how we accounted for the impact of other disease ($\gamma =0$ to $\gamma =1$).

Recall that $\gamma$ is a discount factor that represents the impact on other diseases when newly-acquired ICU beds (*B*(*a*)) are allocated to COVID-19 to the detriment of other non-COVID severe illnesses

When $\gamma = 0$ and EVPI equals zero or is very small, it means that there is no need to gather more information before acting. When $\gamma =0.8$, there is a significant consequence of acting without collecting further information. In such case, if the cost of collecting further information before acting is less than the financial or health cost of 65 ICU beds, the management should invest in resolving the parameter ($R_0$, *CS* and *CI*) uncertainties before deciding which action (number of ICU beds to purchase for COVID-19) to take.

For a given prior belief in either low, medium, or severe outbreak (Fig. 2), the EVPI varies for a fixed $\gamma$. At $\gamma = 0.8$, *belief in severe outbreak* has the highest EVPI. This means that there is more value in collecting further information about other severe non-COVID disease before determining the number of ICU beds to acquire for COVID-19. Information gathering is less important, if there is a *belief in medium outbreak* and even less important when there is a *belief in low outbreak*. However, the EVPI of 33 ICU beds for *belief in low outbreak* is substantial and thus further information to resolve parameter uncertainties could be sought if the cost of such information is lower than the financial or health cost of 33 ICU beds. When there is a maximum concern about non-COVID severe illness ($\gamma = 1)$, then *belief in severe outbreak* has the highest EVPI of 91 ICU beds. This means that further information will be valuable to confirm this belief before choosing the best available management action.

The results show that the value of information increases as the excess allocation discounting factor, $\gamma$, increases until a peak is reached. This trend is consistent across all scenarios. Only *belief in severe outbreak* increases EVPI continuously with $\gamma$ until $\gamma = 1$. Hence, the value of learning about the severity of COVID-19 is affected by our belief that other diseases will also be severe. Conversely, if one believes that excess allocation of ICU beds to COVID-19 will increase the severity of other pre-existing diseases, then there is greater value in learning if COVID-19 will actually be severe in order to ensure optimum allocation of ICU beds without excesses.

We note that when $\gamma =0$, the $EVPI = 0$. If the impact of other non-COVID conditions do not matter in a country, the best action is to purchase or allocate the maximum available ICU bed to COVID-19. There is no value in seeking further information about the nature of an outbreak.

### Expected value of partial information and per parameter EVPI

The decision to gather more information depends on the value of the EVPI being *‘high enough’*. The threshold for a high enough EVPI depends on the context and on the risk preference of health managers. If the EVPI is deemed high, collecting more information is desirable. In this case, we may want to determine which parameter is most important to collect information about. EVPXI per scenario and parameter-level EVPI help us to prioritise information collection at the scenario and parameter levels.

Figure 2 shows that the prior *belief in severe outbreak* has large EVPI across $\gamma > 0$. We, therefore, use this prior of *belief in severe outbreak* to study which scenario and parameters we should be resolving first when there are inadequate resources to resolve all other scenarios and parameters uncertainty.

Table 5 presents the EVPXI at $\gamma ={1}$ and assuming *belief in severe outbreak*. Scenario 17 has the highest value of information and therefore should be resolved first. It also shows a number of scenarios where EVPXI equals zero. These zero-value scenarios belong mostly to where the parameters take *Low* values. That means that there is no value in gathering information on these scenarios before acting. A second cluster is seen where EVPXI are less than 4 ICU beds. We consider these scenarios to have low value of information. For these two groups of scenarios, management could continue without resolving uncertainty for these scenarios. However, for the third cluster the EVPXI range from 5 to 10 ICU beds, it is reasonable to assess that there will be a significant impact of acting without gathering further information about the state of the parameters that define the scenarios in this cluster. The third cluster contains the scenario where no parameter assumes *Low* value.

**Table 5:** EVPXI at $\gamma ={1}$ and with a prior strong belief in severe outbreak: Scenario 17 has the highest VoI and therefore should be resolved first

	Parameter States				Belief in severe outbreak
Scenario ID	*p*(*s*)	$R_0$	Severity (*CS*)	ChildInf (*CI*)	EVPXI ($\gamma = 1$)
1	0.008	Low	Low	Low	0.00
2	0.012	Low	Low	Medium	0.00
3	0.020	Low	Low	High	0.00
4	0.012	Low	Medium	Low	0.00
5	0.018	Low	Medium	Medium	0.00
6	0.030	Low	Medium	High	0.00
7	0.020	Low	High	Low	0.00
8	0.030	Low	High	Medium	0.00
10	0.012	Medium	Low	Low	0.00
11	0.018	Medium	Low	Medium	0.00
12	0.030	Medium	Low	High	0.00
13	0.018	Medium	Medium	Low	0.00
19	0.020	High	Low	Low	0.00
20	0.030	High	Low	Medium	0.00
21	0.050	High	Low	High	0.00
22	0.030	High	Medium	Low	1.00
25	0.050	High	High	Low	1.60
14	0.027	Medium	Medium	Medium	1.80
27	0.125	High	High	High	2.40
15	0.045	Medium	Medium	High	3.8
9	0.050	Low	High	High	4.00
16	0.030	Medium	High	Low	4.00
26	0.075	High	High	Medium	4.00
24	0.075	High	Medium	High	4.90
23	0.045	High	Medium	Medium	5.20
18	0.075	Medium	High	High	6.40
17	0.045	Medium	High	Medium	10.63

Scenario 17 represents the case where $R_0$, case severity and child infectiousness are *Medium*, *High* and *Medium*, respectively (see Tables 4 and 5). There is high value in resolving this scenario first, which means that resolving if this scenario is occurring or not would have the highest gain in management outcomes in comparison with other scenarios. However, due to the dependence in these computation methods, after each high value scenario is resolved to be actually true or false, the most uncertain scenario could change. We cannot determine if scenario 18 (second highest EVPXI) should be resolved next until we first resolve scenario 17. The EVPXI will need to be re-computed before the next scenario with the highest expected value of information is determined.

**Fig. 3:**
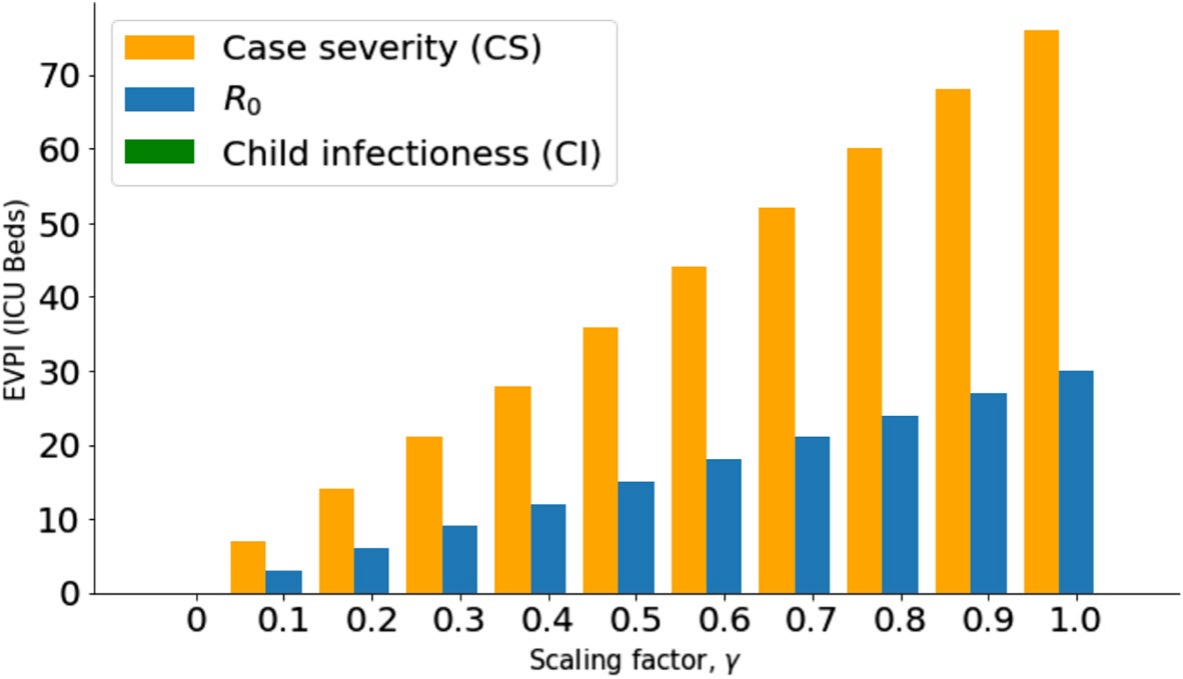
EVPI for *CS*, $R_0$ and *CI* parameters: For all scaling factors, $\gamma$, case severity for an outbreak has the highest value of information, then $R_0$. There is zero value for resolving only the uncertainties surrounding Child infectiousness for all $\gamma$

If there are inadequate resources to collect more information about all three parameters that constitute each scenario, then EVPXI cannot tell us which parameter we should focus our research on. To solve this problem, we conducted EVPI on separate parameters. Figure 3 shows the value of resolving the uncertainty surrounding each of the three parameters without having the knowledge of the other two parameters.

**Fig. 4:**
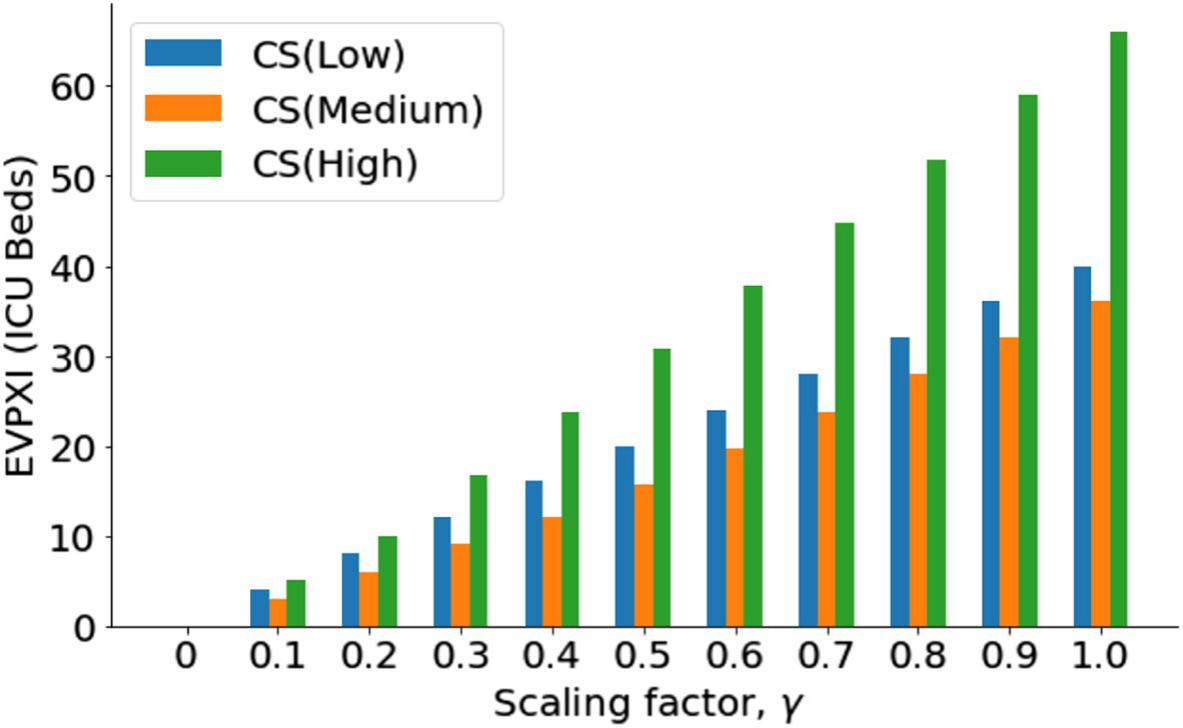
EVPXI for case severity (Low, Medium and High): For all $\gamma$, resolving if case severity is High has the highest value of information followed by if it is Low

Figure 3 shows that case severity is the parameter that mostly drives the value of information for optimum ICU bed acquisition. The excesses and shortages are determined by the case severity of COVID or non-COVID diseases in a country ($\gamma$). This is explained in Fig. 4 using EVPXI for case severity (Low, Medium and High).

**Fig. 5:**
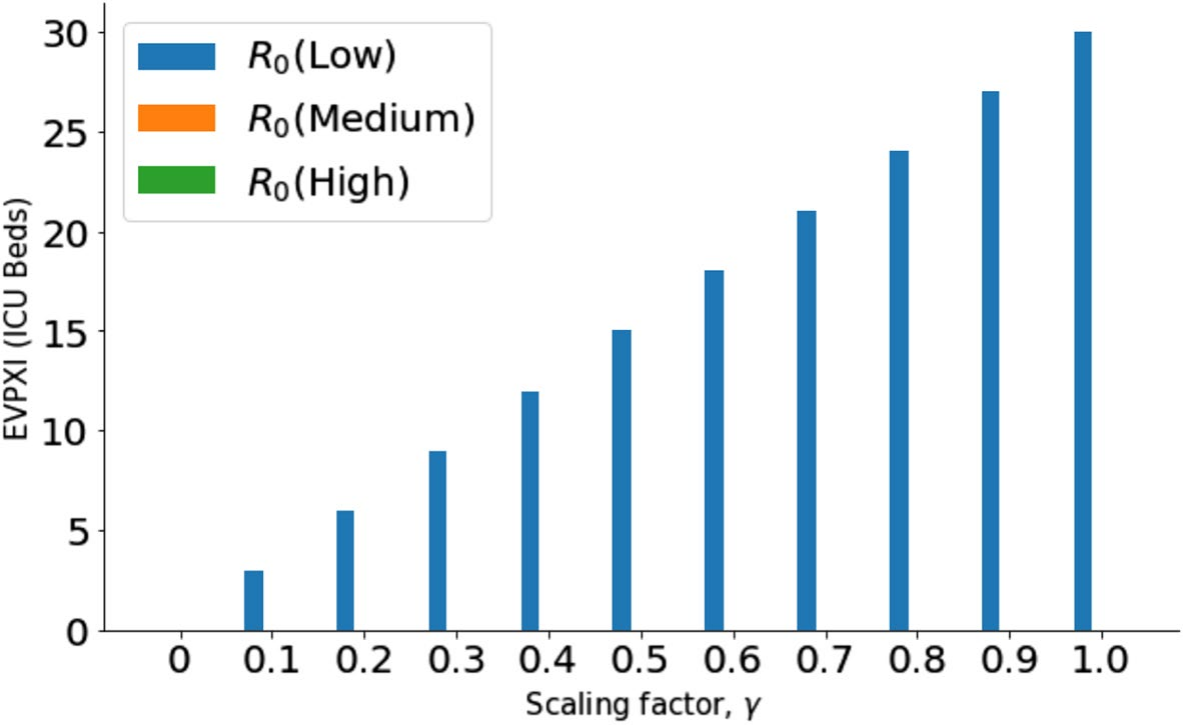
EVPXI for $R_0$ (Low, Medium, High): Resolving if $R_0$ is *Low* has the highest value of information for all $\gamma$. Resolving if $R_0$ is High or Medium is not required before the decision on the number of ICU beds to be acquired for COVID and non-COVID infections

From Fig. 4, the highest research priority is resolving if the case severity of disease outbreak is high or not. With no sufficient resources to conduct research about all the severity levels, a binary test on *High Severity* will reduce the uncertainty surrounding the optimum allocation of ICU beds between COVID and non-COVID patients. The EVPXI will then be computed again for low case severity (*CS*(*Low*)) or medium case severity (*CS*(*Medium*)) to determine which hypothesis will be resolved next. Similar analysis can be performed for $R_0$. The outcome of similar analysis for $R_0$ is shown in Fig. 5.

Finally, an equivalent analysis for Child Infectiousness shows zero value for all its possible states (Low, Medium, or High). Therefore, if case severity and $R_0$ are known, management action will not change when we obtain new knowledge about child infectiousness. Thus, there is no need to spend any research funds on studying child infectiousness before deciding on optimal ICU bed allocation.

## Discussion

We have investigated how VoI analysis can help guide COVID-19 outbreak preparedness when considering the requirement for ICU beds in the early stages of an outbreak.

Our results suggest that reducing uncertainty about case severity and $R_0$ were most valuable because increased knowledge of these factors would have the greatest impact on a decision about how many ICU beds were required. In contrast, reducing uncertainty about child infectiousness was less likely to affect a decision about ICU bed requirements. Hence, in the context of this case study, VoI analysis could enable a manager to make more informed decisions about where they allocate resources in the early stages of an outbreak. Spending resources on studying child infectiousness before deciding on how many ICU beds should be allocated to COVID-19 is unnecessary, as it will not affect the decision outcomes. Spending resources on resolving severity and transmissibility, on the other hand, could affect a decision outcome about investment in ICU capacity. Hence, this study illustrates how mathematical modeling and VoI analysis can provide evidence to enable health managers to decide how resources should be allocated, leading to better decisions about outbreak preparedness.

Note that our analysis was conditioned on the specific question that was being addressed about ICU capacity, and does not mean that the relative infectiousness of children is never a valid factor to consider in preparing for an outbreak. If the question concerns other forms of preparedness, such as providing general ward beds and health workers, VoI analysis may well reveal that child infectiousness is an important factor to be resolved.

To put this work into practical context and in line with existing literature, we consider financial and health costs associated with public health decisions during a COVID-19 outbreak. In financial context, an ICU bed unit costs $37,500 (range: $25,000 - $50,000) [28] to be purchased and installed. So, taking *belief in severe outbreak* and $$\gamma = 0.7$$ as an example, we have EVPI $$=$$ 69 ICU beds which would cost $2.59 million. However, human health and life are not directly measured in dollars. The chosen health cost of information is the number of deaths and other COVID-19 morbidity that will result from a shortfall of 69 ICU beds for patients that may require them. Hence, considering health cost, a unit shortage in ICU beds could increase the mortality rate of the initial strain of COVID-19 by 0.0034% of a given population [14, 29] in a non-vaccinated population. Based on our study objective, having perfect information about an outbreak helps in optimal allocation of ICU beds to avert this loss, which can be significant as this projection means that 34 people in every one million could die. This benefit of optimal allocation, irrespective of the outbreak scenario, is equivalent to the dollar cost or health cost of (not having) 69 ICU beds when they are required for either COVID or non-COVID severe cases.

From the cost analysis above, we can see that VoI analysis helps to explain why the choices made by the manager are the best given the information available at the time. This justification is necessary in order to account for possible losses or gains in the long run. For this study, VoI could help to explain that $2.59 million needs to be spent on purchasing ICU beds in order to increase the chance of saving 34 people per 1 million of the population from dying from a severe COVID-19 infection. For the population size considered in this study (about half a million) and for the $$\gamma = 0.7$$, only half of the dollar cost ($1.29 million) should be spent.

VoI analysis is an integral part of the adaptive management process in fields such as ecology [3, 9] and public health management [12] . Adaptive management is a structured and iterative process that incorporates uncertainty for robust decision-making. A new iteration is required when new information becomes available through a monitoring and feedback process. As more information about an outbreak becomes available, VoI analysis helps public health managers to refine their management actions to optimise health outcomes. When VoI is high, the expected benefit of gathering further information is justified - triggering an adaptive management process to gather the new evidence that can be used to review management actions [3]. The required information can be obtained by carrying out further research in the literature or setting up experiments to obtain the current values of the parameters. This new information may result in a different choice of action that will lead to a different outcome than the action chosen before new information became available.

In the early outbreak of an infectious disease when not much is known about the disease, mathematical modeling helps one to study the dynamics of the disease. Mathematical models enables the study of *what-if* or hypothetical scenarios to understand certain characteristics of the disease such as duration, peak time, transmissibility, severity and the impact of various possible interventions. Mathematical modeling, therefore, provides the expert information when there is no existing information from experts in the outbreak. Also, mathematical models reduce the burden of assembling numerous experts in order to provide a distribution for various parameters instead of relying on a single data point. In our case study, we can see how we utilised models to generate the expert input used in our VoI analysis. Beyond this input generation, mathematical models have become an invaluable tool in predicting, assessing and controlling potential outbreaks [21, 30].

In our case study, we chose to use the shortfall of ICU beds as a proxy for the costs associated with a severe COVID-19 outbreak. An alternative would be to quantify the economic costs associated with not having sufficient ICU beds, for example, using Quality-adjusted life years (QALY) or Disability-adjusted life years (DALY). Note also that while the case study used in this paper has focused solely on preparedness in terms of ICU bed capacity, there are many management decisions required for pandemic preparedness and response. Other preparedness includes stocking prophylactic drugs, recruitment of more health workers and quarantine services, among others [29, 31]. However, the general approach proposed in this paper can be applied to a broad range of decisions involved in the pandemic response.

A potential limitation of our work is that in our exemplar scenario, we focused on three uncertain parameters: severity, child infectiousness and $R_0$. It is possible that model parameters for which we assumed fixed values, such as the latent period, may also affect model outcomes. While the values we used for these fixed parameters were based on the best available evidence at the time, the impact of uncertainty in these parameters remains to be explored. However, the focus of our work is on an example of how to utilise VoI for preparedness for a novel respiratory disease outbreak, with COVID used as a case study.

### Supplementary Information

**Additional file 1.**

## Data Availability

An Excel sheet for one-off computation of VoI as used in this work is available at https://github.com/KingPeter2014/VOIProject/blob/main/VoICUDataMultivariate2.xlsx. The code for advanced and extended analysis can be found at https://github.com/KingPeter2014/VoIAnalysis.git.

## Appendix

This paper contains a Supplementary information on the parameters and methods used for the simulation and clinical pathway models.

## Abbreviations

COVID: Corona Virus Diseases
VoI: Value of Information
EVPI: Expected Value of Perfect Information
EVPXI: Expected Value of Partial Perfect Information
ICU: Intensive Care Unit
SEIR: Susceptible-Exposed-Infectious-Recovered
LHS: Latin Hypercube Sampling
