# Supplementary material for: Value of information analysis for pandemic response: intensive care unit preparedness at the onset of COVID-19
Source: BMC Health Serv Res. 2023 May 13;23:485. doi: 10.1186/s12913-023-09479-4 (PMC10182758; doi:10.1186/s12913-023-09479-4)
Supplement: Supplementary file 1 — Additional file 1. [file 12913_2023_9479_MOESM1_ESM.docx]

**Value of Information analysis for pandemic response: intensive care unit preparedness at the onset of COVID-19**

**Supplementary Information**

*Peter U. Eze, Nicholas Geard, Christopher M. Baker, Patricia T. Campbell and Iadine Chades*

**1.0 Age Stratification and associated risk of severe infection**

***Table S1:*** *Age associated risk of severe outcomes at baseline, and for the two sensitivity analyses accounting for co-morbidities in the population*

| Age group  (years) | Baseline  Severity=1 | Severity = 2 from 30 years |
| --- | --- | --- |
| 0–4 | 0.00062 | 0.00062 |
| 5–9 | 0.00062 | 0.00062 |
| 10–14 | 0.00062 | 0.00062 |
| 15–19 | 0.00062 | 0.00062 |
| 20–24 | 0.00775 | 0.00775 |
| 25–29 | 0.00775 | 0.00775 |
| 30–34 | 0.02900 | 0.09895 |
| 35–39 | 0.02900 | 0.09895 |
| 40–44 | 0.05106 | 0.15493 |
| 45–49 | 0.05106 | 0.15493 |
| 50–54 | 0.09895 | 0.35762 |
| 55–59 | 0.09895 | 0.35762 |
| 60–64 | 0.15493 | 0.65937 |
| 65–69 | 0.15493 | 0.65937 |
| 70–74 | 0.35762 | 0.65937 |
| 75+ | 0.65937 | 0.65937 |

Health experts and epidemiologists from the region are engaged to elicit their belief (in probabilistic terms) in the age-stratified risk for an outbreak **[1]**. Also, COVID-19 is novel and thus, has different pathogenic characteristics in different populations and demographics **[2]** according to early information from the disease dynamics. Hence, all other scenarios might be the actual nature of a potential outbreak in different regions. With VoI analysis, we strive to find out if we could do something differently if the state of nature (defined by severity, Ro and child infectiousness) changes from current information. A major decision problem is selecting which level of preparedness to pursue based on budget and the anticipated values of the epidemiological parameters under study.

***2.0 Infectious Disease Modelling and Simulation***

We adapted the infectious disease transmission model (TM) and clinical pathway model (CPM) first used in [3] to incorporate the experts’ opinion and estimates required in generating **V (a, s)** for VoI analysis. These models provided the cost of a COVID-19 outbreak as the Case severity (CS), Child Infectiousness (CI), and Basic Reproduction Number (R**_0_**) varies from Low, Medium to High. To balance between quantitative and qualitative analysis of these parameters, they were each stratified into three levels: ***{(R0, Severity, Child Inf) = (Low|Medium|High, Low|Medium|High, Low|Medium|High)}***. The quantitative meaning of these tuples is explained in the main paper, yielding the 27 scenarios modeled. The sampling method used is the improved stratified Monte-Carlo method called Latin Hypercube Sampling (LHS) [4].

The disease transmission model [3] estimated how varying the key parameters affects the disease trajectory. This transmission model is a compartmental model in which individuals in a population are separated into different compartments or states based on their health status with respect to the pathogen under study. Here we implemented an SEIR model with individuals Susceptible (S), Exposed (E), Infected (I) or Removed (R). Except for a single infectious individual, the entire population begins the simulation in the S-compartment. Contact between susceptible and infectious people that is sufficient for transmission to occur results in movement from the S-compartment to the E-compartment. The exposed population becomes infectious and moves to the I-compartment at a rate that is the inverse of the duration of the latent period. The infectious population moves to the R-compartment at a rate that is the inverse of the duration of the infectious period. We assumed a latent period of 3.2 days, and an infectious period of 9.68 days.

The CPM shown in ***Figure S1*** was originally developed for influenza [3] and now adapted for COVID-19. The CS parameter applies to this clinical pathway model. From ***Table S1***, the value of CS determines the proportion of cases that become severe within the population and age-group. Only severe cases get hospitalized and only a fraction of the hospitalized cases ends up in the ICU unit. Hence, the higher the value of CS, the higher the proportion of people that are likely to require ICU care. The proportions described in [3] was utilized in this research because the early symptoms of COVID-19 was closer to a flu infection. This is denoted as **ƞ**(eta). This transformed parameter **ƞ** is drawn from a log-uniform distribution that is parameterized by some minimum and maximum exponential values according to equation (1):

$$ƞ\left( x;a,b \right)= {10}^{b} x b<0;0<x<1 (1)$$

where ***b*** is the maximum value for the exponent of the log distribution. The parameter *x* in equation (1) is the probability of the age group needing an ICU bed. The older age group has higher values for *x* than the younger age group, corresponding to higher severity level. The values of ***b*** chosen for the simulation to compute $ƞ$ using equation (1) are shown in **Table** **S2** below. The sixteen age groups and sample severity levels associated with each age group are shown in Table **S1.**

***Table S2****:* ***Severity parameters for calculating Hospitalized proportion for each severity level***

|  | Low value | Medium value | High value |
| --- | --- | --- | --- |
| Case Severity (CS) | 1 | 2 | 3 |
| *b* | -3 | -2 | -1 |

The selection is such that between 5 to 30% of the infected population are hospitalized depending on the age-group. Then, a fixed proportion of the hospitalized patients end up in the intensive care unit (ICU). The exact proportion of the hospitalized proportion is determined by the CS parameter (Low, Medium or High) of the outbreak. With the parameter values now chosen and explained for the three major parameters under study, it is time to run the models to generate the outputs that will be utilized in the VoI analysis.

***
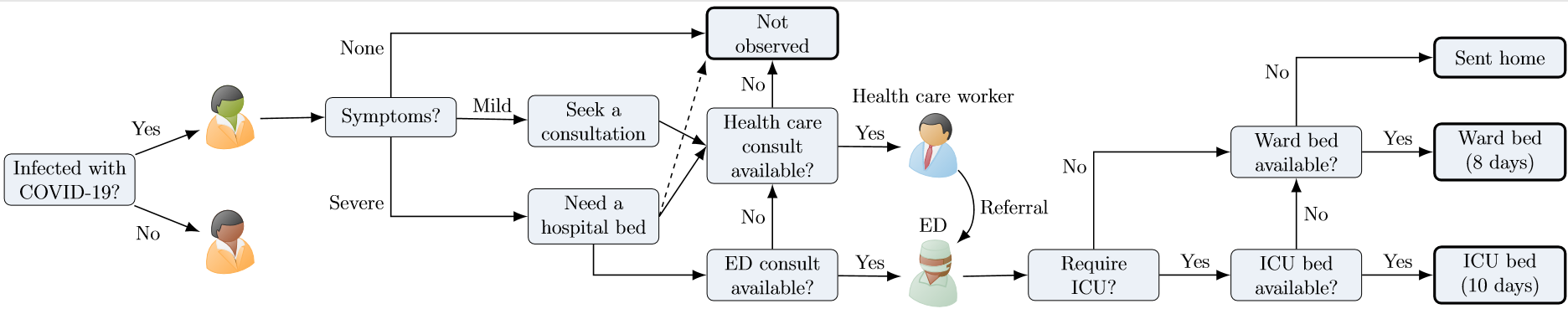
***

***Figure S1:*** *Clinical pathways model from Moss et al [3] showing the flows of patients with mild and severe disease through available services in the health care system*

***2.1 Simulation Procedure and Parameter Analysis***

To explain how the variation in the parameter value translates into model parameters, we incorporate some code snippets from our MATLAB model . One of the parameters is selected and varied from Low to Medium to High while keeping the other two variables within either the low, medium or high strata. For example, when we vary case severity (**CS**), CI and R_0_ will remain at its constant strata while CS varies. As CS is not sampled from a range, it means that we run the severity scenarios with say {(**CS =1**, CI= [High], R_0_ = [Medium]), (**CS =2**, CI= [High], R_0_ = [Medium]), (**CS =3**, CI= [High], R_0_ = [Medium])}.

Hence, the cost (payoff), Vs, for s = 1…27, associated with each of the 27 hypotheses is given by:

$$X_{s}=\prod_{R0, Low}^{High} \prod_{CI,Low}^{High} {TM}_{R0,CI} \prod_{CS,Low}^{High} {CPM}_{CS} (8a)$$

***TM*** is the transmission model which requires R_0_ and Child Infectiousness (CI) to run so that the number of infected individuals will be generated per unit time in a population. The infected individuals are now passed through the Clinical Pathway Model (***CPM***) and depending on the proportion of severe cases, the CPM generates the number of individuals that will likely require an ICU bed. Based on this generated value and on the initial number of ICU beds available before the outbreak, the extra ICU bed requirement is computed. The sum of these excesses over the duration of the outbreak serves as the cost, **X_s_** for the scenario,.


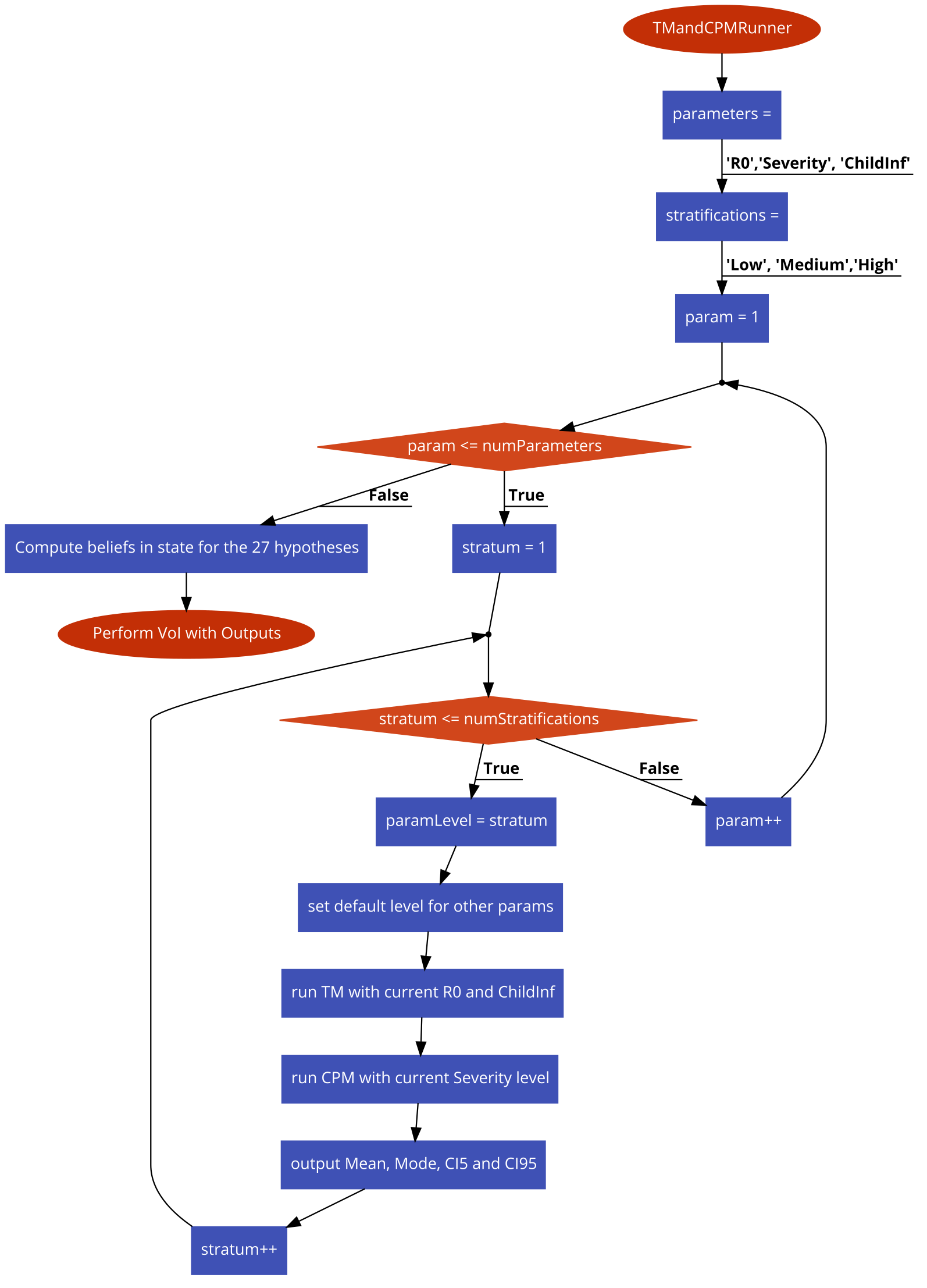


***Figure S2***: Flowchart for the simulations that generated the costs utilized for VoI Analysis.

Depending on the value assigned to ***CS***, the proportion of severe cases is drawn according to the a log-flat (uniform) probability distribution. A positive random variable X is log-uniformly distributed if the logarithm of ***X*** is uniform distributed. The probability density function of a reciprocal distribution, from which a log-uniform distribution is derived, is given as:

$f\left( x;a, b \right)= \frac{1}{x\left[ \log_{e} b- \log_{e} a \right]} for a \leq x \leq b;a>0 (8c)$

Taking the logarithm (to any base) of the reciprocal distribution gives a uniform distribution.

For Child Infectiousness (CI), we use a uniform distribution to randomly select the value that will be applied to the proportion of transmission that occurs among the children (aged 0 – 19) when they interact. The values are drawn from a uniform distribution according to the limits in **Table 3**.

Hence, depending on the value of “currentStrata”, values will be randomly assigned to Child infectiousness from either the Low, Medium or High range.

To generate the cost/payoff required for the VoI analysis, the transmission model is executed first and its output in terms of Infectious individuals are fed into the Clinical pathway model [3]. The Clinical Pathway Model (CPM) illustrates the flow of patients through the health system. At each node in the pathway, it assesses whether consultation or admission is possible within the available daily capacity. It also assesses what kind of admission (Ward or Critical care) is required. In this way, it can estimate periods during which ICU capacity is exceeded over the course of the epidemic. This result helps us to determine the different levels of preparedness for ICU beds depending on the possible outbreak scenarios. The Daily Presentations (number of people who came to seek medical attention in hospitals) and the Excess ICU demand (extra ICU requirement per day) over a 365-day outbreak scenario were computed and recorded. The Excess ICU demands were summed over the period to compute the Net Excess ICU demand (the extra ICU beds per year that health managers need to purchase to supplement the existing ones, if any) for the duration of the outbreak. This simulation was carried out for the 27 hypotheses (states of nature). For each hypothesis simulation, each parameter making up a hypothesis is sampled 200 times within its defined range to establish the confidence intervals. These confidence intervals are utilized as the payoffs (cost in our case) for each of the actions or decision alternatives available to health the managers. The cost or payoff is represented by **V (a, s)** in the VoI analysis. The summary of the code snippet used to perform each simulation is shown below. The provided code is run for each of the hypothesis using a Monte-Carlo LHS of 200 parameter samples per scenario.


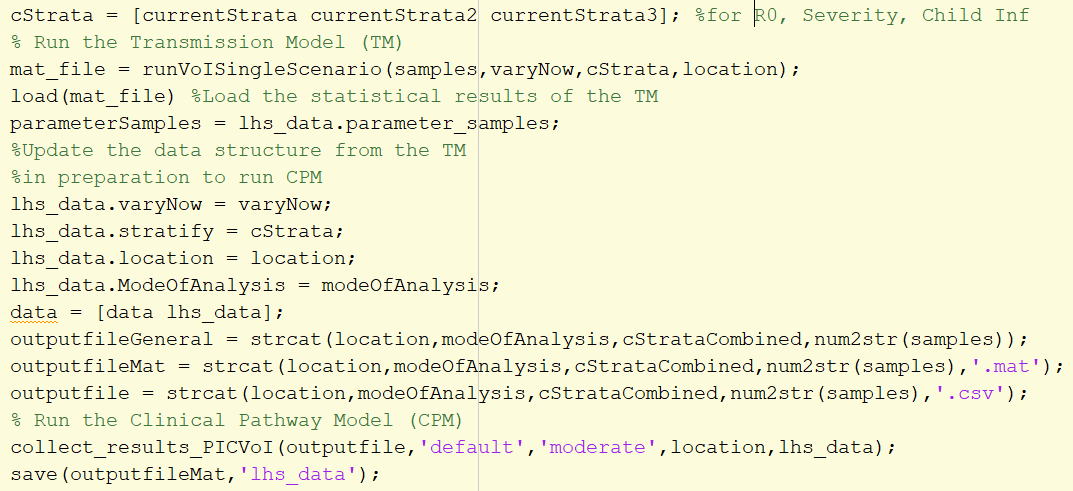


**3.0 Summary of the Clinical Pathway Model Output**

The different combinations of case severity, transmissibility and child infectiousness lead to different outcomes in terms of excess ICU demand, X(s).

**
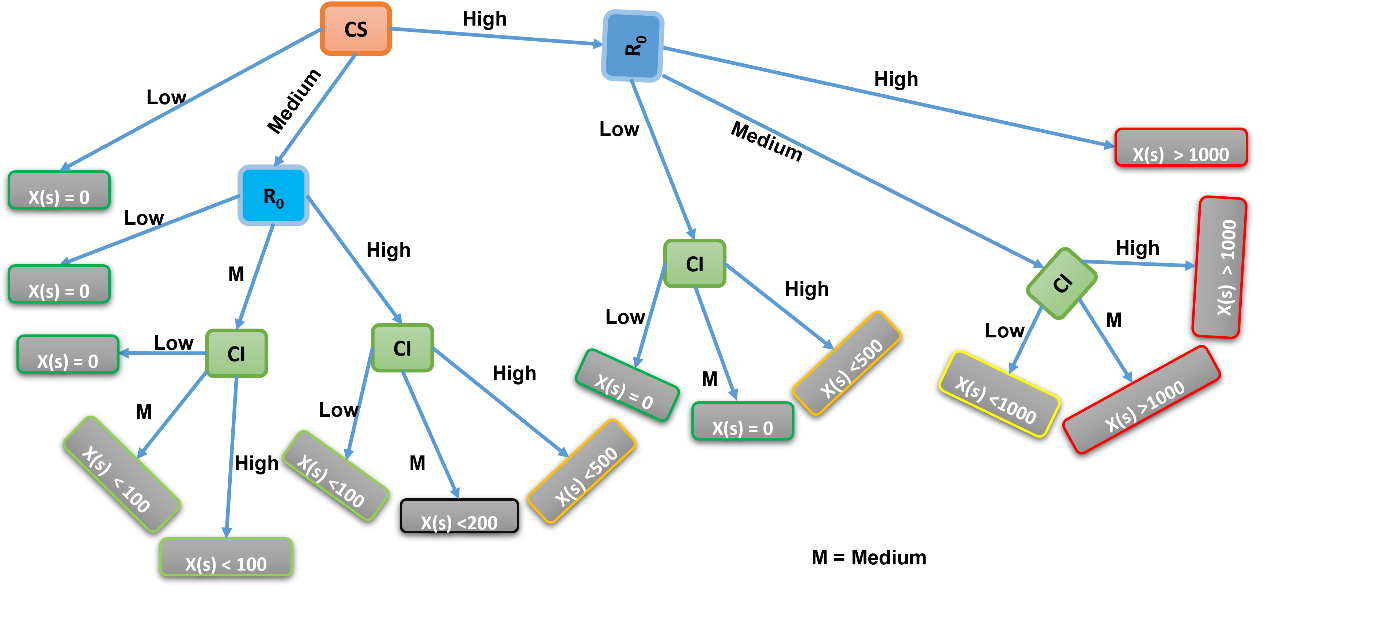
**

**Figure S3:** A decision tree showing the combination of parameter values and their outcome in terms of number of Excess ICU demand, X(s).

**References**

[1] Nicol S., Ward K., Stratford D., Joehnk K.D and Chades I. ***Making the best use of experts’ estimates to prioritise monitoring and management actions: A freshwater case study***. Journal of Environmental Management, vol. 215, pp. 294 – 304, 2018. Doi: 10.1016/j.jenvman.2018.03.068.

[2] Hu B., Guo H., Zhou P. et al. ***Characteristics of SARS-CoV-2 and COVID-19***. Nat Rev Microbiol (2020). <https://doi.org/10.1038/s41579-020-00459-7>.

[3] Moss R., McCaw J. M., Cheng A. C., Hurt A. C., and McVernon J. “***Reducing Disease Burden in an Influenza Pandemic by Targeted Delivery of Neuraminidase Inhibitors: Mathematical Models in the Australian Context***.” BMC Infectious Diseases 16, no. 1 (October 10, 2016): 552. <https://doi.org/10.1186/s12879-016-1866-7>

[4] Imam, R. ***Latin Hypercube Sampling***. Online, Online (1999). <https://doi.org/10.1002/9780470061596.risk0299>
